# Diagnostic stewardship applied to targeted fungal sequencing

**DOI:** 10.1128/jcm.00896-25

**Published:** 2025-08-29

**Authors:** Jessica Hudson, Indre Budvytiene, Niaz Banaei

**Affiliations:** 1 Department of Pathology, Stanford University School of Medicine 10624 , Stanford, California, USA; 2 Clinical Microbiology Laboratory, Stanford Health Care, Palo Alto, California, USA; 3 Division of Infectious Diseases & Geographic Medicine, Stanford University School of Medicine 10624 , Stanford, California, USA; University of Calgary, Calgary, Alberta, Canada

**Keywords:** diagnosis, stewardship, sequencing, fungal, tissue

## LETTER

Accurate and rapid diagnosis of invasive fungal disease (IFD) is challenging due to the lack of sensitivity of fungal culture and nonspecificity of histopathology (1, 2). Targeted fungal PCR followed by amplicon sequencing of the ribosomal RNA genes and internal transcribed spacers is routinely performed on fresh and formalin-fixed paraffin-embedded (FFPE) tissue to accurately diagnose IFD and tailor antifungal therapy (3, 4). Recommended testing criteria include sterile samples with evidence of fungal elements, which is consistent with the definition of proven IFD outlined by the European Organization for Research and Treatment of Cancer and the Mycoses Study Group Education and Research Consortium (EORTC/MSGERC) (4–7). The aim of this study was to investigate the outcomes of patients whose fungal sequencing requests were canceled due to the absence of fungal elements on histopathology.

**Fig 1:**
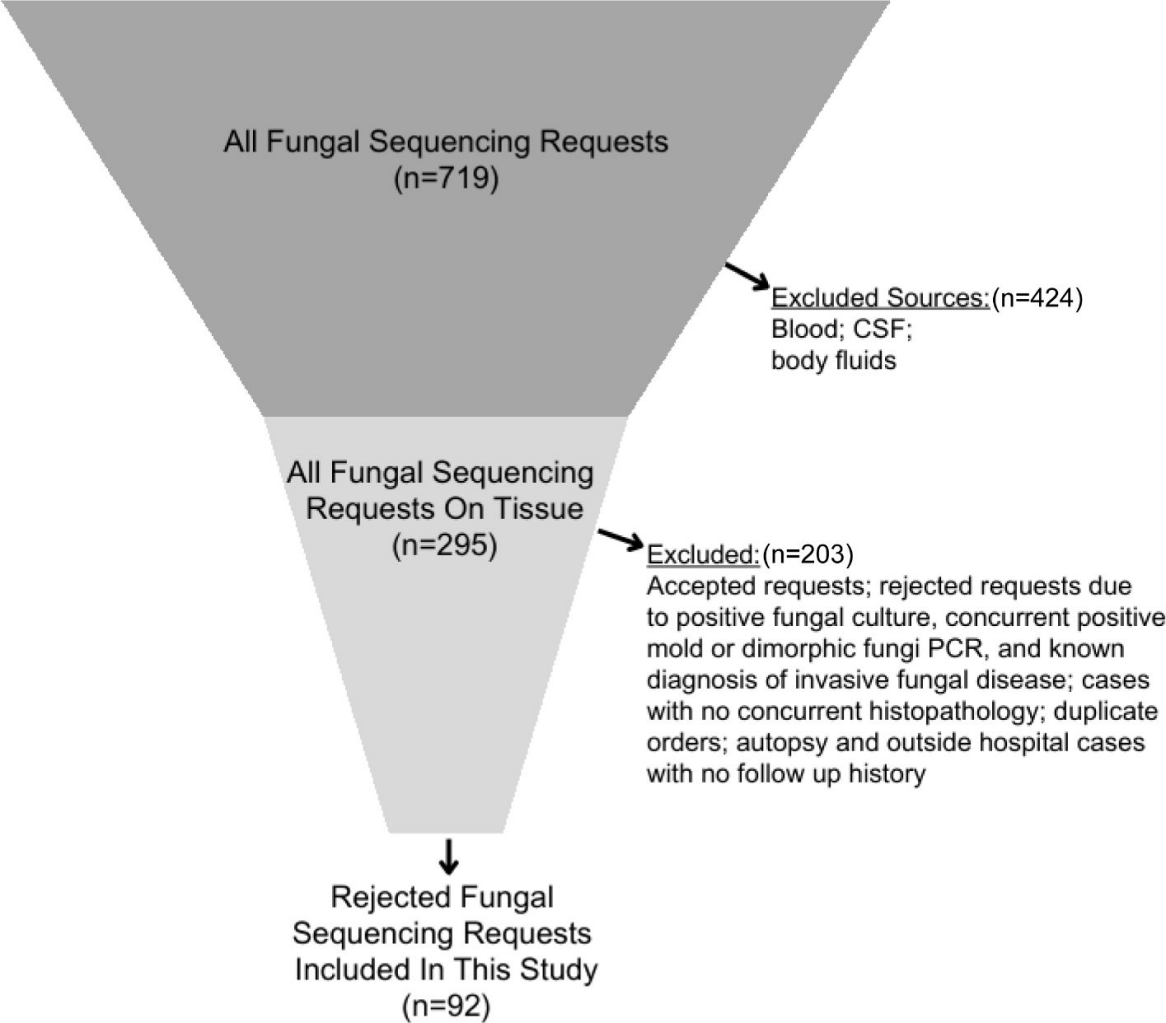
Funnel chart showing schematic overview of fungal sequencing requests and inclusion of rejected fungal sequencing requests in this study. Fungal sequencing acceptance criteria included (i) tissue from a sterile source, (ii) tissue with fungal elements on concurrent histopathology or ancillary evidence of positive antigen test for dimorphic fungi, (iii) tissue without mixed organism morphologies on microscopy, (iv) absence of growth in culture, and (v) known diagnosis of invasive fungal disease. Rejected fungal sequencing requests on 10 fresh and 82 formalin-fixed paraffin-embedded samples were included in this study based on the absence of fungal elements on concurrent histopathology.

From January 2018 through February 2025, 719 fungal sequencing requests were reviewed to identify canceled orders due to the absence of fungal elements on concurrent histopathology (Fig. 1). Acceptance and rejection criteria for fungal sequencing are shown in Fig. 1. Among the orders accepted, 100% had evidence of fungal elements on histopathology. Chart review was performed to obtain demographic, clinical, laboratory, and outcome data. The key objective was to determine if IFD or an alternative diagnosis was made during the follow-up period. This study was a quality improvement project, and approval from the institutional review board was waived.

Ninety-two patients with canceled fungal sequencing orders (10 fresh and 82 FFPE tissue) were included in this study (Table S1). At the time of biopsy, 39 (42.3%) were immunocompromised and based on the EORTC/MSGERC case definitions, 0 had proven, 1 (1.1%) had probable, 19 (20.7%) had possible, and 72 (78.3%) had no IFD. Eleven patients had died at the time this study was conducted. Zero patients were diagnosed with IFD during the follow-up period of 3 months to 7 years. In 74 (80.4%) patients, alternative diagnoses were established during follow-up, including 32 (43.2%) bacterial infections, 15 (20.2%) malignancies, and 7 (9.4%) autoimmune diseases. Sixteen tissues had granulomatous inflammation in which Grocott’s methenamine silver and/or Periodic Acid-Schiff with diastase stains were performed in 15 and all were negative. Among the 16 tissues with granulomatous inflammation, 5 (31.3%) were associated with malignancy, 3 (18.7%) with mycobacterial infection, and 2 (12.5%) with chronic granulomatous disease. During the follow-up period, one patient was identified with a noninvasive fungal process characterized as chronic allergic fungal sinusitis (8).

This study provides direct evidence to support the cancellation of fungal sequencing on tissue without fungal elements on the corresponding histopathology. We showed among 92 patients with canceled fungal sequencing requests due to the absence of fungal elements on corresponding histopathology, none developed IFD during the follow-up period. In other words, the negative predictive value of absence of fungal elements on histopathology was 100% for ruling out IFD. This finding is particularly reassuring given that 16 patients had granulomatous inflammation, which is a known immunopathological response to dimorphic fungi and a reason to perform fungal sequencing in the absence of fungal elements. Yet, none had or developed IFD. Findings of this study are also consistent with prior studies showing the highest yield of fungal sequencing is in tissues with fungal elements on corresponding histopathology (9, 10). Avoiding fungal sequencing in low-risk patients and on samples with low pretest probability for IFD is critical for saving resources and mitigating false-positive results due to environmental contamination or detection of non-infectious flora. This practice may be best achieved by asking providers and pathologists to only request fungal sequencing in patients with host and clinical risk factors for IFD based on the EORTC/MSGERC definitions and on samples with evidence of fungal elements, respectively. Furthermore, the molecular microbiology laboratory director and staff must also practice diagnostic stewardship and make sure that only samples that meet criteria are tested. However, in cases when clinical suspicion remains high for IFD, further sampling may be needed to diagnose IFD.

The limitations of this study included a moderate number of samples from a single institution. Therefore, findings might not be generalizable to centers with different patient populations, surgical pathology practices, and sequencing assays.

## SUPPLEMENTAL MATERIAL

Table S1 jcm.00896-25-s0001.docx Chart review findings.

ASM does not own the copyrights to Supplemental Material that may be linked to, or accessed through, an article. The authors have granted ASM a non-exclusive, world-wide license to publish the Supplemental Material files. Please contact the corresponding author directly for reuse.
