# Supplementary material for: Diagnostic stewardship applied to targeted fungal sequencing
Source: J Clin Microbiol. 2025 Aug 29;63(10):e00896-25. doi: 10.1128/jcm.00896-25 (PMC12506030; doi:10.1128/jcm.00896-25)
Supplement: Table S1 — Chart review findings. [file jcm.00896-25-s0001.docx]

**Supplementary Table 1.** Demographics and clinical characteristics of patients included in this study


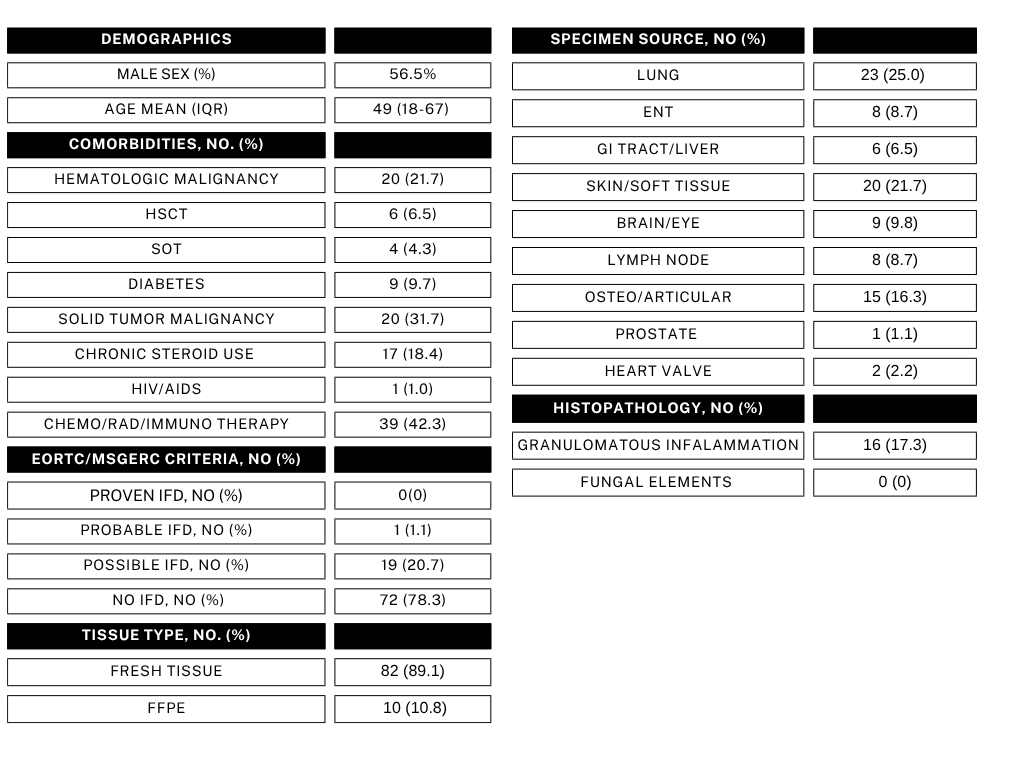


IQR, interquartile range; NO., number; HSCT, hematopoietic stem cell transplant; SOT, solid organ transplant; HIV, human immunodeficiency virus; AIDS, acquired immunodeficiency syndrome; CHEMO, chemotherapy; RAD, radiation; EORTC/MSGERC, European Organization for Research and Treatment of Cancer and the Mycoses Study Group Education and Research Consortium; IFD, invasive fungal disease; FFPE, formalin-fixed paraffin-embedded; ENT, Ear, Nose, and Throat, GI, gastrointestinal
